# A review on the function of arabinogalactan-proteins during pollen grain development

**DOI:** 10.1007/s00497-024-00515-9

**Published:** 2025-02-06

**Authors:** Sara Foubert-Mendes, Jessy Silva, Maria João Ferreira, Luís Gustavo Pereira, Sílvia Coimbra

**Affiliations:** 1 LAQV/REQUIMTE, Biology Department, Faculty of Sciences, University of Porto, Porto, Portugal; 2 School of Sciences, University of Minho, Campus de Gualtar, Braga, Portugal; 3 GreenUPorto-Sustainable Agrifood Production Research Centre/INOV4AGRO, Biology Department, Faculty of Sciences, University of Porto, Porto, Portugal

**Keywords:** Arabinogalactan-proteins, Pollen development, Pollen tube, Exine, Intine

## Abstract

**Key message:**

Overview of the current understanding of PG development, PT growth and the role of AGPs in these processes.

**Abstract:**

The pollen grain (PG) is a complex structure composed of three cells: the vegetative cell which develops into a pollen tube (PT) and two sperm cells that will fuse with the egg cell and central cell, giving rise to the embryo and endosperm, respectively. This resilient gametophyte is constantly subjected to selective pressures, leading to a diverse range of characteristics, with one of its defining features being the pollen cell wall. In this review, we closely examine the developmental stages of PG formation and PT growth, with a specific focus on the dynamic roles of arabinogalactan-proteins (AGPs) throughout these processes. AGPs are initially present in pollen mother cells and persist throughout PT growth. In the early stages, AGPs play a crucial role in primexine anchoring, followed by nexine and intine formation as well as cellulose deposition, thereby providing essential structural support to the PG. As PGs mature, AGPs continue to be essential, as their absence often leads to the collapse of PGs before they reach full maturity. Moreover, the absence of AGPs during PT growth leads to abnormal growth patterns, likely due to disruptions of cellulose, callose, and F-actin deposition, as well as perturbations in calcium ion (Ca^2+^) signalling. Understanding the intricate interplay between AGPs and PG development sheds light on the underlying mechanisms that drive reproductive success and highlights the indispensable role of AGPs in ensuring the integrity and functionality of PGs.

## Introduction

In a dynamically changing environment, where the forces of natural selection drive species adaptation and evolution, it is important for organisms to generate genetically diverse progeny. Genetic variability is primarily achieved through sexual reproduction. In embryophytes, the sexual life cycle alternates between the haploid gametophytic and diploid sporophytic generations. Sporophytes produce microspores and megaspores, which subsequently develop into male and female gametophytes, respectively (Yadegari and Drews 2004).

In angiosperms, the male gametophyte, or pollen grain (PG), is a highly specialised structure designed to deliver male haploid cells to unfertilised ovules through the pollen tube (PT), thus giving rise to the next generation. The resilient gametophyte is under constant selective pressure from environmental factors, such as the type of pollination and the abundance of pollinators, as well as from interactions with its female counterparts, or even interactions among other male gametophytes. This has resulted in a remarkably diverse set of characteristics unique to PGs, which become further specialised during their germination into a pollen tube.

Arabinogalactan proteins (AGPs), a family of highly glycosylated proteins, are believed to be key players in the reproductive process. AGPs are implicated in various plant developmental processes, including cell–cell communication, signalling, and structural integrity of the cell wall (Seifert and Roberts 2007; Ma et al. 2018). Recently, the capacity of AGPs to work as calcium (Ca^2+^) reservoirs is helping to solve the role of these important glycoproteins in several fundamental biological processes, possibly because calcium is an essential ion for plant development (Lamport and Várnai 2013). Despite these significant advances in understanding AGP functions, their precise roles during pollen development remain elusive. Since the beginning of our studies, we have been showing AGPs sugar epitopes to be tissue and developmental stage specific, from the early stages of pollen mother cell formation through to PT growth, but the mechanisms by which they influence these processes are not fully understood. Recent studies have highlighted the importance of AGPs in primexine anchoring, nexine and intine formation, as well as in cellulose deposition, which are essential for maintaining PG integrity and facilitating successful PT growth (Jia et al. 2015; Lin et al. 2022).

In this comprehensive review, we present an overview of PG development and PT growth, with a particular focus on the role of AGPs in these processes, using *Arabidopsis thaliana* as the model organism. By integrating findings from various studies, we seek to clarify the multifaceted functions of AGPs during male gametophyte development, highlighting their critical contributions to reproductive success in angiosperms. This synthesis of recent research not only underscores the importance of AGPs in pollen development but also identifies key areas for future investigation to advance our understanding of these enigmatic proteins.

## Arabinogalactan proteins

AGPs are non-enzymatic proteins belonging to the superfamily of cell wall hydroxyproline-rich glycoproteins (HRGPs) together with extensins and proline-rich proteins. AGPs are generally distinguished from other HRGPs by their high density of *O-*linked glycosylation of hydroxyproline residues. Furthermore, their protein core is also rich in serine, alanine, and threonine. Usually, most of the molecular weight of AGPs is due to their carbohydrate moiety, frequently around 90% (w/w), whereas the protein core represents only around 10% (w/w). The carbohydrate moiety is composed of type II arabinogalactan (AG) polysaccharides, which consist of a (1–3)-linked β-D-galactose backbone and small (1–6)-linked β-D-galactose side chains, usually modified at the *O*-3 and *O*-6 positions with arabinose. Further modifications of the side chains with other sugars, such as L-rhamnose, D-mannose, D-xylose, D-glucose, L-fucose, and D-glucuronic acid can also be present, creating a large variability among the AGP family. In addition to the high percentage of amino acids PAST (proline, alanine, serine, and threonine) and abundant *O-*glycosylation of hydroxyproline residues, the presence of an N-terminal cleavable signal peptide that targets AGP precursors to the endoplasmic reticulum (ER) is another key property of AGPs. In addition, most AGPs contain a hydrophobic C-terminal domain, which is cleaved in the ER to promote the addition of a glycosylphosphatidylinositol (GPI) lipid anchor that tethers the AGP molecules to the outer leaflet of the plasma membrane (Silva et al. 2020).The properties of the polypeptide core, such as its composition and the presence of particular motifs, distinguish the different classes of AGPs, namely classical AGPs, with a PAST percentage greater than 42% and a length of more than 90 amino acid residues; AG-peptides, with 55–90 amino acid residues; lysine-rich (Lys-rich) AGPs with a Lys-rich domain between the proline-rich and the C-terminal domains; chimeric AGPs include fasciclin-like AGPs (FLAs) with fasciclin-like domains, early nodulin-like AGPs (ENODLs, also known as phytocyanin-like AGPs) that have plastocyanin-like domains, and xylogen-like AGPs (XYLPs) with non-specific lipid transfer protein (nsLTP) domains (Showalter et al. 2010; Ma et al. 2017; Johnson et al. 2017). Additionally, Ma et al. (2017) suggested that the chimeric AGP class should also include subclasses based on the presence of special domains, namely protein kinase-like, forming homology 2-like, glycosyl hydrolase-like, pollen Ole e I-like, leucine-rich repeat-like, X8-like, pectin methylesterase inhibitor-like, pectate lyase-like, and SGNH hydrolase-like domains. Hybrid AGP-extensins (HAEs) are often considered to belong to a class of their own (Johnson et al. 2017). The evolutionary conservation of AGPs across such a wide range of plant species suggests essential roles in plant growth, development, reproduction, and possibly in adaptation to environmental challenges.

AGPs are widely distributed throughout the plant kingdom, as shown by positive reactions with β-Yariv reagent and immunolocalisation studies (Seifert and Roberts 2007; Ma et al. 2018). The β-Yariv reagent is a synthetic chemical compound which binds specifically to the β-(1 → 3)-linked D-Gal*p* backbone of AGPs, precipitating them (Yariv et al. 1967) and making it a valuable tool to study the biology of AGPs. In immunolocalisation studies, the use of monoclonal antibodies that bind specifically to AGP sugar epitopes have also been an invaluable tool to map the presence of different AGP populations in various plant tissues and developmental stages. Several monoclonal antibodies have been developed to target AGP sugar epitopes, each with specific binding properties, such as JIM8, JIM13, or LM2 (Yates et al. 1996; Ruprecht et al. 2017), but because of the likely common basic structure of glycan chains in different AGPs, this approach cannot be used to distinguish between specific gene products.

Several studies in *Arabidopsis* have revealed that AGPs are essential for pollen development and PT germination and growth (Table 1), including AGP6 (AT5G14380), AGP11 (AT3G01700), AGP40 (AT3G01700), AGP50, also known as BCP1 (AT1G24520), FLA3 (AT2G24450), and FLA14 (AT3G12660) (Xu et al. 1995; Levitin et al. 2008; Coimbra et al. 2009; Li et al. 2010; Nguema-Ona et al. 2012; Miao et al. 2021). Also, the presence of AGPs have been reported during pollen development in plants other than *Arabidopsis*, such as those in the *Brassica* genus or in rice (Table 1).

**Table 1:** Information about the AGPs expressed in the male reproductive tissues and presumed functions in several species

Species	AGP	Locus	Expression	Mutant Phenotypes	Function(s)	References
*Arabidopsis thaliana*	AtAGP6	At5g14380	PGs; PTs	Collapsed pollen grains devoid of content (*agp6 agp11*); low PG germination and shorter PTs (*agp6 agp11*); reduced seed set and precocious PT germination (*agp6 agp11 agp40*)	PG development; Nexine layer formation; PT growth	Levitin et al. (2008); Coimbra et al. (2009); Coimbra et al. (2010); Nguema-Ona et al. (2012); Jia et al. (2015)
	AtAGP11	At3g01700	PGs; PTs		PG development; PT growth	Levitin et al. (2008); Coimbra et al. (2009); Coimbra et al. (2010); Nguema-Ona et al. (2012)
	AtAGP23	At3g57690	PGs; PTs	–	–	Pereira et al. (2014)
	AtAGP24	At5g40730	PGs; PTs	–	–	Moreira et al. (2022)
	AtAGP40	At3g20865	–	Reduced seed set and precocious PG germination (*agp6 agp11 agp40*)	PG germination	Nguema-Ona et al. (2012)
	AtBCP1/AGP50	At1g24520	PGs	Non-viable and aborted PGs (antisense RNA)	PG development	Xu et al. (1995)
	AtFLA3	At2g24450	PGs; PTs	Shorter filaments, PT guidance, and fertility defects (OE); Shorter filaments, withered anthers, aborted PGs, and thinner, uneven intine layer, abnormal cellulose deposition, and low PG germination (RNAi)	Microspore development; Intine formation; PG germination	Li et al. (2010)
	AtFLA14	At3g12660	PGs	Precocious PG germination in high moisture; Collapsed PGs devoid of content and abnormal intine layer and cellulose deposition (OE)	Microspore development; Intine formation; PG germination	Miao et al. (2021)
*Brassica campestris*	BcMF8	Bra000995	–	Collapsed PGs and low PG germination (antisense RNA)	PG development	Lin et al. (2014); Lin et al. (2018)
	BcMF18	Bra008762	–	Collapsed PGs, lower stigma adhesion of PGs, and low PG germination (antisense RNA); Collapsed PGs and low PG germination (OE)	PG development	Lin et al. (2018); Lin et al. (2019)
*Brassica rapa*	BrFLA2	Bra001464	PGs; PTs	Precocious PG germination in high moisture [*fla2 fla28 fla32* (RNAi)]	PG germination	Huang et al. (2021)
	BrFLA28	Bra034746	PGs; PTs			
	BrFLA32	Bra038741	PGs; PTs			
*O. sativa* ssp. *indica*	OsIAGP	OsIFCC010429	PGs; PTs	–	–	Anand and Tyagi, (2010)
*O. sativa* ssp. *japonica*	OsMTR1	OS02G28970	PGs	Incorrect tapetum vacuolation, reduced sporopollenin precursor, delayed tapetum PCD, slow microspore growth, microspores unable to perform mitosis and aborted PG, which led to male sterility	Tapetum functioning; Microspore development	Tan et al. (2012)
*Gossypium hirsutum x G. barbadense*	GhFLA19	GH_D12G1039	PGs	Short filaments, shriveled anthers, delayed tapetum PCD and aborted PGs with a thin intine and irregular exine	Tapetum functioning; PG development; Intine and exine formation	Zhang et al. (2021)

OE, overexpression; PG, pollen grain; PCD, programmed cell death; PT, pollen tube; RNAi, RNA interference

## AGPs in male gametophyte development

**Fig. 1:**
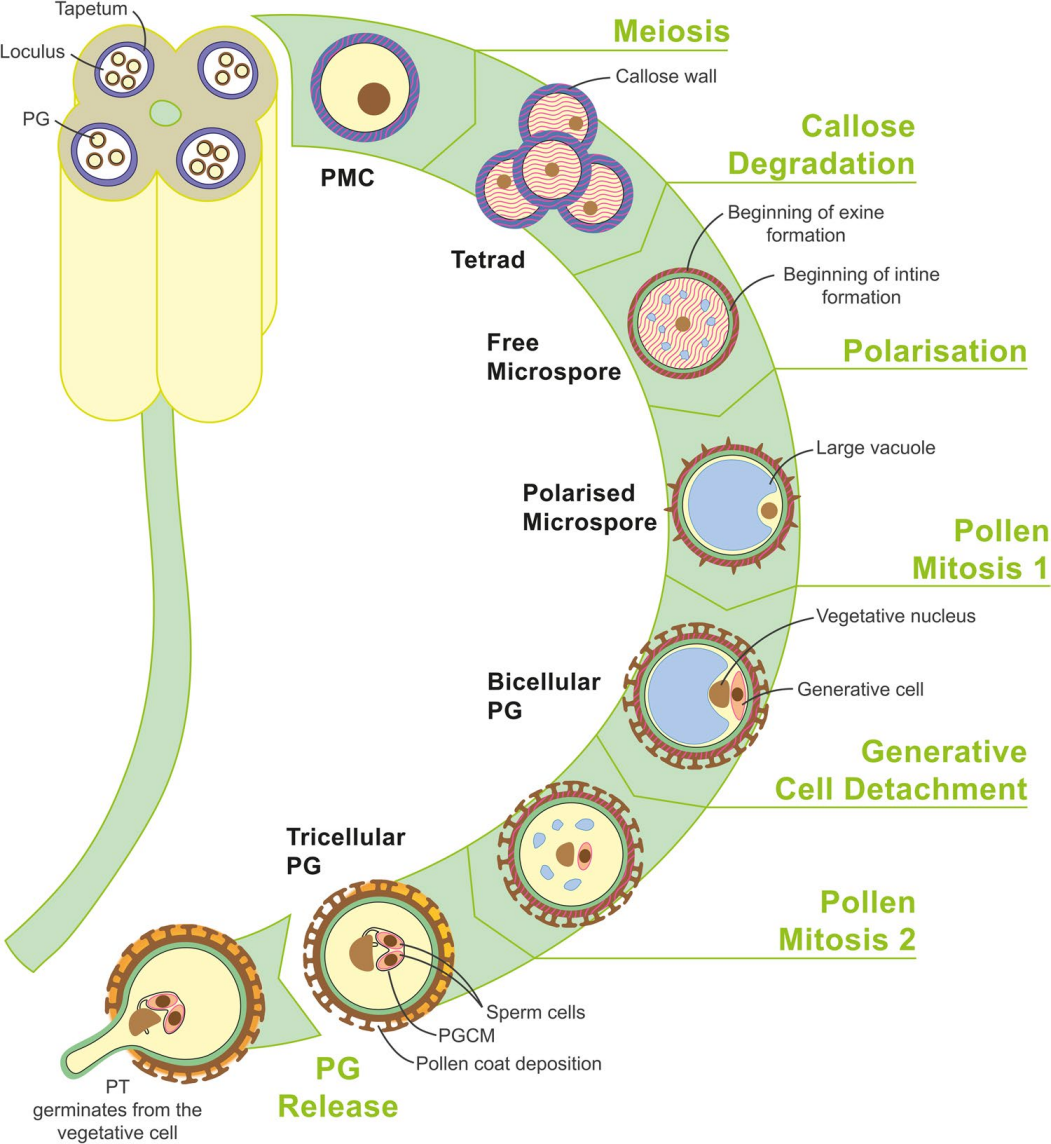
Male gametophyte development inside the anther in *A. thaliana*. The male gametophyte develops inside the anthers, which are composed of several tissues and cell layers, including the tapetum that encloses the loculi where gametophytes will develop. During microsporogenesis, a diploid pollen mother cell (PMC) undergoes meiotic division to produce a tetrad of four haploid microspores surrounded by a callose wall. AGPs (pink lines) are initially detected in PMC cell walls. After meiosis, AGPs appear in the cytoplasm and primary cell walls of tetrads. Microspores are released by degradation of callose by callase secreted by tapetal cells and formation of intine and exine layers is initiated. Free microspores contain AGPs within the exine cavities and in the cytoplasm. During microgametogenesis, microspores polarise, enlarge, and undergo an asymmetric division, pollen mitosis 1, which results in a bicellular pollen grain (PG) with a small generative cell engulfed within the cytoplasm of a large vegetative cell. AGPs remain in the exine and are also present in the generative cell wall. The vegetative cell exits the cell cycle, and the generative cell detaches from the wall of the vegetative cell and undergoes further mitotic division, pollen mitosis 2, to produce two sperm cells (tricellular PG). AGPs are also detected in the cell walls of the sperm cells. The sperm cells, which are connected by a common extracellular matrix, are enclosed by the peri-germ cell membrane (PGCM). One sperm cell has a cytoplasmic projection that directly links to the vegetative cell nucleus, forming the male germ unit (MGU). Finally, tapetum degradation promotes the deposition of the pollen coat, and mature pollen is released and lands on the stigma, where it hydrates and germinates into a pollen tube from the vegetative cell, which grows through the style to deliver the sperm cells into the female gametophyte. Abbreviations: PG, pollen grain; PGCM, peri-germ cell membrane; PMC, pollen mother cell; PT, pollen tube

Male gametophyte development is divided into two phases: microsporogenesis and microgametogenesis (Fig. 1). Both processes occur within the anther locules, which are surrounded by the tapetum, a fundamental tissue for pollen development and release. During microsporogenesis, diploid pollen mother cells (PMCs), derived from the primary sporogenous layer within each anther locule, undergo meiosis, resulting in the formation of a tetrad of haploid microspores. Each microspore is enclosed in a thick callose (β-1,3-glucan) layer, synthetised by callose synthases of PMCs and later by callose synthases of the microspores themselves. As microspore development progresses, callose is degraded by the enzyme callase, which is secreted by the tapetal cells (Borg et al. 2009; Gómez et al. 2015; Halbritter et al. 2018; Adhikari et al. 2020a; Hafidh and Honys 2021). A change in the AGP expression pattern becomes evident from the early stages of the male gametophyte development. In *Arabidopsis* and with the aid of monoclonal antibodies, AGPs first appear in the cell walls of PMCs (JIM8) and endothecial cells (JIM8 and JIM13). After meiosis, AGPs were found in the cytoplasm and primary cell wall of tetrads (JIM8), forming a reticulated layer above the protectum of tetrads (MAC204) as well as in the locule-facing cell wall of the tapetum (JIM8 and MAC204) (Suzuki et al. 2017).

Upon degradation of the callose layer, microspores separate from each other, and microgametogenesis begins with the polarisation of microspores, during which a large vacuole pushes the nucleus against the cell wall (Borg et al. 2009; Gómez et al. 2015; Halbritter et al. 2018; Adhikari et al. 2020a; Hafidh and Honys 2021). *Arabidopsis* free microspores show AGPs inside of the exine cavities (JIM8 and MAC204) and the walls of tapetal cells (JIM8, JIM13, and MAC204). AGP labelling was also observed in the cytoplasm and cell wall of microspores (JIM13) (Coimbra et al. 2007; Suzuki et al. 2017). Upon microspore polarisation, the first pollen mitosis follows, generating two asymmetric cells: a large vegetative cell and a small generative cell. This structure is called the bicellular PG (Borg et al. 2009; Gómez et al. 2015; Halbritter et al. 2018; Adhikari et al. 2020a; Hafidh and Honys 2021). During this stage, in *Arabidopsis*, AGPs remain in exine cavities between the baculae (JIM8, JIM13, and MAC204) and were observed in the cell wall of the generative cell (JIM8 and JIM13) (Coimbra et al. 2007; Suzuki et al. 2017).

The generative cell then detaches from the vegetative cell wall and moves inside the cytoplasm of the vegetative cell (Borg et al. 2009; Gómez et al. 2015; Halbritter et al. 2018; Adhikari et al. 2020a; Hafidh and Honys 2021). Then, a symmetric division of the generative cell, the second pollen mitosis, results in the formation of two sperm cells that remain connected by a common extracellular matrix. The physical association between the sperm cells and vegetative cell nucleus forms a functional unit named male germ unit (MGU). As a result of this unique developmental process, a specific membrane surrounds the male germ cells; the generative cell and eventually the two sperm cells. In order to standardise and unify, this membrane structure was named as the peri-germ cell membrane (Sugi et al. 2024). The presence of these two sperm cells inside the cytoplasm of the vegetative cell constitutes the mature or tricellular PG (Dumas et al. 1985; Borg et al. 2009; McCue et al. 2011; Sprunck 2020; Sugi et al. 2023) and AGPs were also found to be present in the cell walls of *Arabidopsis* sperm cells (JIM8 and JIM13) (Coimbra et al. 2007; Suzuki et al. 2017).

The first study demonstrating that AGPs sugar epitopes are specific to PGs and PTs, as well as developmentally regulated, was conducted on *Arabidopsis* using the antibodies JIM8, JIM13, and MAC207 (Coimbra et al. 2007). In addition to *Arabidopsis*, innumerable immunohistochemistry studies have been performed to detect AGP epitopes in various species, such as *Brassica napus*, *Oryza sativa*, *Zea mays*, *Secale cereale*, *Quercus suber,* and many others (Ma et al. 2018). Following this study, several others were conducted as an attempt to identify which AGPs could present these specific epitopes. Results obtained using qPCR and reporter lines in *Arabidopsis* revealed that specific AGPs, namely *AGP6*, *AGP11*, *AGP23* (AT3G57690), *AGP24* (AT5G40730), and *FLA3*, were highly expressed in PGs and PTs, indicating their importance in reproductive tissues (Coimbra et al. 2008; Pereira et al. 2014; Moreira et al. 2022). *AGP23* was found to be specific to the vegetative cell and *AGP24* was highly expressed in pollen as well as in female reproductive tissues (Pereira et al. 2014; Moreira et al. 2022). *FLA14* has also been found in PGs (Miao et al. 2021).

In *B. napus*, AGPs were predominantly identified in the cell walls of tetrads and in later stages in the cytoplasm and vesicles of the vegetative cell (JIM14) (El-Tantawy et al. 2013) or conspicuously distributed internally and around the generative cells (JIM13, LM2, and LM6). Interestingly, MAC207 was the only antibody to label the intine of the polarised microspore and the cytoplasm and cell wall of mature PGs, especially at the aperture sites (El-Tantawy et al. 2013). In contrast, a recent study revealed that AGPs in *B. napus* were mostly detected by JIM13 in the intine of microspores (Corral-Martínez et al. 2019).

In *O. sativa*, JIM8 and JIM13 labelling were detected in the cytoplasm of PMCs and later in tetrads alongside anther walls throughout development (Ma et al. 2019). In mature PGs, JIM8, JIM13, and LM2 labelling localised to the cell wall, whereas the MAC207 signal appeared in both the cytoplasm and pollen wall (Ma et al. 2019).

In *Q. suber*, JIM8 and JIM13 recognised AGP epitopes in the cell wall of PMCs during meiosis and tapetum excreted vesicles (Costa et al. 2015). A signal was later observed in the intine near the aperture sites and in later stages in both the intine and generative cell wall. JIM8, JIM13, and MAC207 labelled both the tapetum and its secretions (Costa et al. 2015). Similarly, in *Trithuria submersa (Hydatellaceae),* JIM8 and JIM13 recognised AGP epitopes in the intine layer, especially at the aperture sites of mature pollen (Costa et al. 2013). In *S. cereale*, AGP epitopes were observed in the cell wall of polarised microspores, encompassing both exine and intine (JIM4 and JIM13). Furthermore, using the same monoclonal antibodies, AGP epitopes were identified in the walls of anthers and in excreted cytoplasmic vesicles of microspores (Zieliński et al. 2021). In *Nicotiana tabacum*, immunolocalisation using JIM13 revealed AGP epitopes in the cell wall of the generative cells (Qin et al. 2007).

AGP6 and AGP11 share a sequence similarity of 75% and an amino acid sequence identity of 68% (Pereira et al. 2006). AGP6 and AGP11 appear to have redundant functions, with AGP6 assuming a dominant role, as its inactivation showed a mutant phenotype that was not visible on *agp11* mutant (Levitin et al. 2008; Coimbra et al. 2009). Analysis of the *agp6 agp11* double mutant revealed a key role of this AGP pair in successful PG development, as over 50% of all PGs appeared to have collapsed and were devoid of content (Coimbra et al. 2009). The PGs of the *agp6 agp11* mutant had lower germination rates and produced shorter PTs than their wild-type (wt) counterparts (Coimbra et al. 2009).

Male sterility with non-viable and aborted PGs has been observed in plants with *AGP50* downregulated by antisense RNA (Xu et al. 1995). Since the observed phenotype was also partially due to the lack of *AGP50* in the tapetum, which is fundamental for PG development, Xu et al. (1995) used the tomato pollen-specific promoter of *LAT52* to drive the antisense *AGP50* RNA, aiming to determine the function of this AGP only in PGs, leaving the expression in the tapetum unperturbed. Diminished *AGP50* levels in PGs led to a 50% rate of aborted PGs. This male sterility phenotype, reminiscent of the *agp6 agp11* mutant, began at the polarised microspore stage in transgenic lines guided by the *AGP50* promoter, and at the bicellular stage in *LAT52* promoter guided lines (Xu et al. 1995).

Several FLAs in *B. rapa* were studied by Huang et al. (2021), namely *BrFLA2* (Bra001464), *BrFLA28* (Bra034746), and *BrFLA32* (Bra038741), using RNA interference (RNAi) technology. These genes are all orthologues of *AtFLA14*, and RNAi lines also caused premature PG germination inside the anthers under high humidity conditions. However, unlike *AtFLA14*, *B. rapa* FLAs are present not only in PGs and PTs, but also in anther tissues (Huang et al. 2021).

## AGPs during pollen wall development

**Fig. 2:**
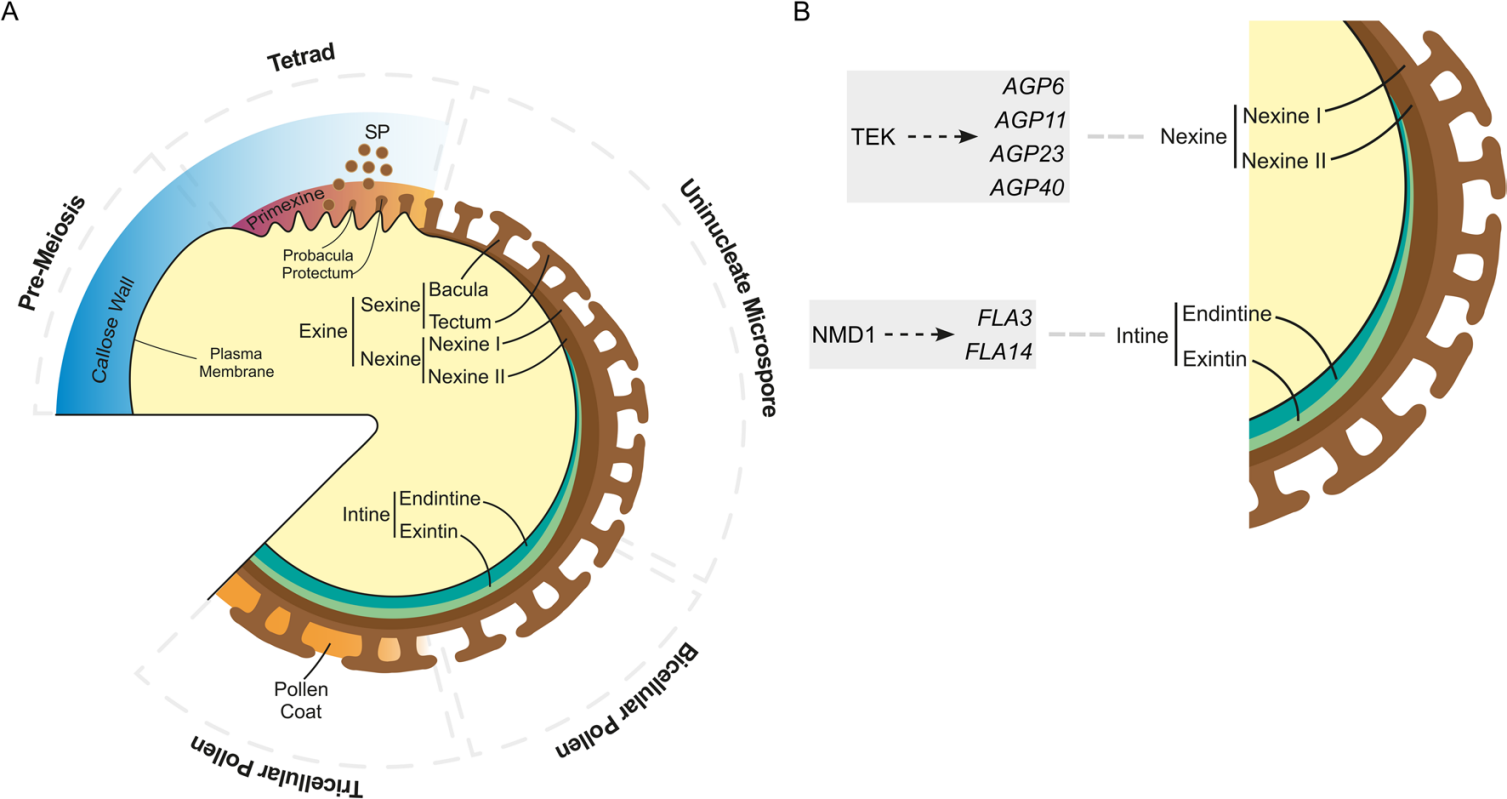
Pollen wall formation and composition in *A. thaliana*. **A** Pollen wall formation is initiated after meiosis in the tetrad stage, when primexine is deposited between the callose wall and the plasma membrane. The plasma membrane gradually becomes undulated. Sporopollenin precursors (SP) secreted by tapetal cells accumulate on the primexine template, forming the probacula and protectum on the undulated plasma membrane. The callose wall and primexine are degraded, undulations of the plasma membrane disappear, and uninucleate microspores are released. The nexine layers, nexine I and nexine II, initiate formation. Tapetal cells continue to secrete material, and the probacula and protectum originate bacula and tectum structures, respectively, which are the constituents of the sexine layer. The intine layers, exintine and endintine, are deposited between the plasma membrane and nexine, and are completely formed at the bicellular pollen stage. The tapetal cells undergo programmed cell death (PCD), and the remnants fill the spaces between the sexine on the pollen surface, forming the pollen coat on the tricellular pollen grain stage. The mature pollen wall is composed of three layers: pollen coat, exine, and intine. The exine is divided into two layers: the inner bilayer nexine, which consists of the nexine I and nexine II, and the outer sexine, which contains the tectum and baculae. The intine is comprised of two layers: exintin and endintine. **B** We hypothesise that TEK regulates (dashed arrow) *AGP6*, *AGP11*, *AGP23*, and *AGP40*, which are crucial for nexine formation and consequently play a key role in normal pollen grain development. On the other hand, NMD1 regulates (dashed arrow) *FLA3* and *FLA14* which are important for intine formation. Abbreviations: SP, sporopollenin

Once the mature pollen stage is reached, the pollen wall, which is an important communication and protection interface, has also completed its formation. It is a complex wall composed of three layers: a pollen coat, an outer layer called exine, composed of sporopollenin, and an inner pectocellulosic layer called intine. Both exine and intine layers are further divided. Exine comprises a sexine layer, subdivided into tectum and bacula, and a nexine layer, subdivided into nexine I, also known as the foot layer, and nexine II. Intine is divided into two layers: endintine and exintin (Fig. 2A).

In rice (*O. sativa* ssp. *indica*)*,* the *OSIAGP* gene (OsIFCC010429), an orthologue of *AtAGP23*, is a pollen-preferential gene that also showed activity during germination and PT growth (Table 1) (Anand and Tyagi 2010). Furthermore, in *O. sativa* ssp. *japonica*, a recessive mutation in the *MICROSPORE AND TAPETUM REGULATOR1* (*OsMTR1*; Os02G28970) gene resulted in a sterile male plant (Table 1). This chimeric AGP shares a high sequence similarity with AtFLA20 (AT5G40940) and is responsible for correct vacuolation of the tapetum at the tetrad stage of pollen development. Its disturbance also caused slower growth of the free microspores, which failed to perform mitosis and were eventually aborted. Simultaneously, the tapetum had smaller and fewer Ubisch bodies (responsible for delivering sporopollenin precursors to the locule), and therefore, did not provide sufficient sporopollenin precursors. Moreover, the tapetum did not fully degenerate in due time (Tan et al. 2012).

Similarly, a sterile male mutant was obtained from *Gossypium hirsutum* and *Gossypium barbadense* hybrid breeding (Table 1). This aberrant phenotype was due to the combination of a recessive trait homologous to *AtFLA19* (AT1G15190) and *OsMTR1*. The deletion of this *GhFLA19* (GH_D12G1039) gene using CRISPR/Cas9 technology resulted in flowers with short stamen filaments and shrivelled anthers, associated with collapsed PGs with a thinner intine and irregular exine. The degeneration of the tapetum was also delayed and several genes involved in pectin catabolic process, actin cytoskeleton organisation, cell tip growth, protein transport, and carbohydrate transport were downregulated, as well as orthologues of genes known to be key in intine formation and fatty acid transporters, which are important for exine development (Zhang et al. 2021). Regarding the *agp6 agp11* phenotype during pollen development, Jia et al. (2015) tried to explain it by relating the collapse of PGs with the formation of nexin, showing that AGP6 was fundamental for the correct formation of this layer.

The synthesis of the pollen wall begins soon after the formation of the callose wall around the tetrads. At the tetrad stage, upon the deposition of a microfibrillar matrix designated primexine between the plasma membrane and callose wall, the plasma membrane changes its texture from smooth to convoluted. Primexine, which is primarily composed of cellulose, polysaccharides, and proteins, plays a key role in guiding the deposition and assembly of sporopollenin precursors. Sporopollenin is derived from tapetal cells and is mostly composed of long-chain fatty acids that are often covalently linked to other molecular groups, particularly phenylpropanoid derivatives. Using the primexine matrix as a guide, the main component of the pollen wall, sporopollenin, accumulates, facilitating proexine development. Proexine contains precursors of the bacula and tectum, named probacula and protectum, respectively. Additionally, primexine guides pollen wall patterns, whereas probacula patterning is modelled according to plasma membrane undulations (Fig. 2A) (Jiang et al. 2013; Shi et al. 2015). Following callose degradation, microspores are liberated into the locule, and primexine is degraded (Lou et al. 2014b). In addition, the nexine layer is established and surrounds the plasma membrane. Simultaneously, due to subsequent material deposition from tapetal cells, the probacula grows and elongates, increasing the size of the sexine layer, which, in conjunction with the nexine, forms the exine (Jiang et al. 2013; Lou et al. 2014b; Shi et al. 2015).

Analysis of a null mutant line for *AGP40,* an AG-peptide, showed normal pollen development but PGs with reduced fitness (Nguema-Ona et al. 2012). An *agp6 agp11 agp40* triple mutant was obtained, which displayed a significant reduction in seed production and a higher number of early PT germination inside the anther than the *agp6 agp11* double mutant (Coimbra et al. 2010; Nguema-Ona et al. 2012). Interestingly, Jia et al. (2015) showed that TRANSPOSABLE ELEMENT SILENCING VIA AT-HOOK (TEK; AT2G42940) can function as a transcription factor for *AGP6*, *AGP11*, *AGP23*, and *AGP40* (Fig. 2B). In addition, *tek* mutants presented male sterility caused by a lack of nexine and intine layers, which are fundamental to prevent PG collapse (Lou et al. 2014a; Jia et al. 2015). Furthermore, *tek* microspores did not undergo mitotic divisions. The authors of this study hypothesised that the lack of the nexine impeded intine formation (Lou et al. 2014a), further implicating the interdependence of pollen wall layers and the presence of AGPs in these layers for normal PG development.

Upon full development of the nexine, the intine layer begins to form under the control of the gametophyte. The intine consists of a granular exintine, containing pectin and protein inclusions, and endintine composed of microfibrillar cellulose. The intine wall is completed when the PG terminates pollen mitosis 1. At later stages of pollen development, the tapetum undergoes programmed cell death and the remains of degenerating tapetal cells, composed mostly of tryphine (a mixture of lipids, proteins, pigments, and aromatic compounds), fill the spaces between the nexine and the sexine, giving rise to the pollen coat (Fig. 2A) (Jiang et al. 2013; Lou et al. 2014b; Shi et al. 2015). Overall, pollen wall patterning is taxon-specific and widely used for species identification (Jiang et al. 2013; Shi et al. 2015).

In addition to classical AGPs and AG-peptides, FLAs, namely *FLA3* and *FLA14*, were shown to be involved in intine formation in *Arabidopsis*. Overexpression (OE) of *FLA3* resulted in fertility issues due to abnormalities in ovules, filaments, and PT guidance. However, RNAi lines showed a 50% abortion rate for PGs, short filaments, and withered anthers. At the polarised microspore stage, a thinner and uneven intine layer was apparent, and in the collapsed PGs, the intine layer was no longer apparent (Fig. 2B). This phenotype was hypothesised to be due to an abnormal cellulose deposition, since aborted PGs lacked fluorescence when stained with calcofluor white compared to wt. Furthermore, PG germination decreased in vitro, but no change in PT length was observed (Li et al. 2010). A knockdown T-DNA line of *FLA14* showed abnormal PG germination under high humidity conditions, whereas *FLA14* OE lines had a phenotype similar to *agp6 agp11*: aborted PGs appeared shrunken with a lack of cell content. *FLA14* OE plants also showed abnormalities in the intine layer at the polarised microspore stage which apparently resulted in collapsed PGs with degraded cytoplasmic content and no intine (Fig. 2B). However, some PGs could develop a thicker intine layer than wt PGs. Moreover, similar to the *fla3* RNAi PGs, *FLA14* OE led to unusual cellulose deposition (Miao et al. 2021).

The regulatory role of AGPs in intine formation was further supported by transcription factors like ARABIDOPSIS NOVEL MICROGAMETOPHYTE DEFECTIVE MUTANT 1 (NMDM1; AT5G09250), shown to control AGPs expression for proper intine formation (Mi et al. 2022) (Fig. 2B). Mutant lines of this transcription factor edited with CRISPR/Cas9 were lethal if the plant was homozygous for the mutation. On the other hand, heterozygous lines and RNAi knockdown lines showed aborted PGs with unusual cellulose deposition and abnormal intine formation. RNA-seq transcripts of these lines revealed several AGPs, including *FLA3*, *FLA12*, *AGP6*, *AGP23*, and *AGP41*, severely downregulated.

In *B. campestris*, orthologues of *AtAGP6* and *AtAGP11*, *MALE FERTILITY 18* (*BcMF18*; Bra008762) and *BcMF8* (Bra000995), respectively, are important for pollen development. *BcMF8* antisense RNA, *BcMF18* antisense RNA, and *BcMF18* OE plants showed a large number of collapsed PGs and a lower PG germination rate, and in the case of antisense *BcMF18* RNA lines, a lower stigma adhesion of PGs. Unlike their *Arabidopsis* counterparts, these defects stemmed from an abnormal intine layer. The *BcMF8* antisense RNA lines revealed an unusual intine formation and a larger number of aperture sites with uneven intine, whereas *BcMF18* antisense RNA and *BcMF18* OE lines demonstrated a lack of cellulose in the cell wall of PGs. In the case of antisense *BcMF18* RNA lines, a complete lack of intine layer was observed (Lin et al. 2014, 2018, 2019). *BcMF18* antisense transgenic plants had defects from the polarised microspore stage onwards, leading to abortion by the bicellular stage. Antisense *BcMF8* RNA plants revealed retardation of PT growth and unstable PTs that were abnormally shaped and could burst with ease (Lin et al. 2014), whereas those of antisense *BcMF18* RNA plants were shorter (Lin et al. 2018). A double antisense RNA mutant of both AGPs was produced, leading to 76.5% abnormal PG formation, including 27.7% aborted PGs with no nuclei or cellulose deposition and 48.8% PGs deformed with a larger aperture site number than expected. As with the single mutants, abnormal intine thickening was observed at the polarised microspore stage, and PG germination rate was lower. These genes appear to be non-redundant, with BcMF8 appearing to be important in intine formation and aperture site number and position, whereas BcMF18 is more specialised in intine formation (Lin et al. 2018).

## AGPs and PT growth

Following the release of dehydrated PGs from anthers, PGs land on the stigmatic papillae. These cells contain specific receptors that can sense ligand molecules attached to the pollen wall, specifically the pollen coat, which determines whether a PG is rejected or able to hydrate and subsequently germinate. This phenomenon is known as recognition, and some plant families have developed important self-incompatibility processes (Yadegari and Drews 2004; Lopes et al. 2019; Adhikari et al. 2020b; Sprunck 2020; Hater et al. 2020; Hafidh and Honys 2021). Upon contact with the stigma, the vegetative cell generates a PT which will transport the MGU to the ovule (Dumas et al. 1985; Borg et al. 2009; McCue et al. 2011; Sprunck 2020; Sugi et al. 2023). During *Arabidopsis* PG germination, AGP labelling was observed at the site of PT emergence (MAC207) and in a ring-like appearance between the aperture site and the tip of the emerging PT (LM2) (Pereira et al. 2006).

When the PT emerges from the PG, it begins its journey as a vehicle for both sperm cells to reach the embryo sac. This arduous expedition begins with the growth of the PT through the stigmatic papillae, then through the extracellular matrix of the transmitting tract, and finally delivering its cargo through the micropylar aperture to an unfertilised ovule, where one sperm cell fuses with the egg cell and the other with the central cell, giving rise to a diploid embryo and a triploid endosperm, respectively. This process is known as double fertilisation and is specific to angiosperms (Yadegari and Drews 2004; Lopes et al. 2019; Adhikari et al. 2020b; Sprunck 2020; Hater et al. 2020; Hafidh and Honys 2021). During PT growth, AGPs were found in the cell walls of sperm cells (JIM8 and JIM13) and at the walls of the growing tip (Pereira et al. 2006; Coimbra et al. 2007). In *Fragaria x ananassa,* AGP epitopes were labelled in PTs, specifically in the apex (JIM13) and a ring-like pattern in the apex and subapical area (MAC207) (Leszczuk et al. 2019).

**Fig. 3:**
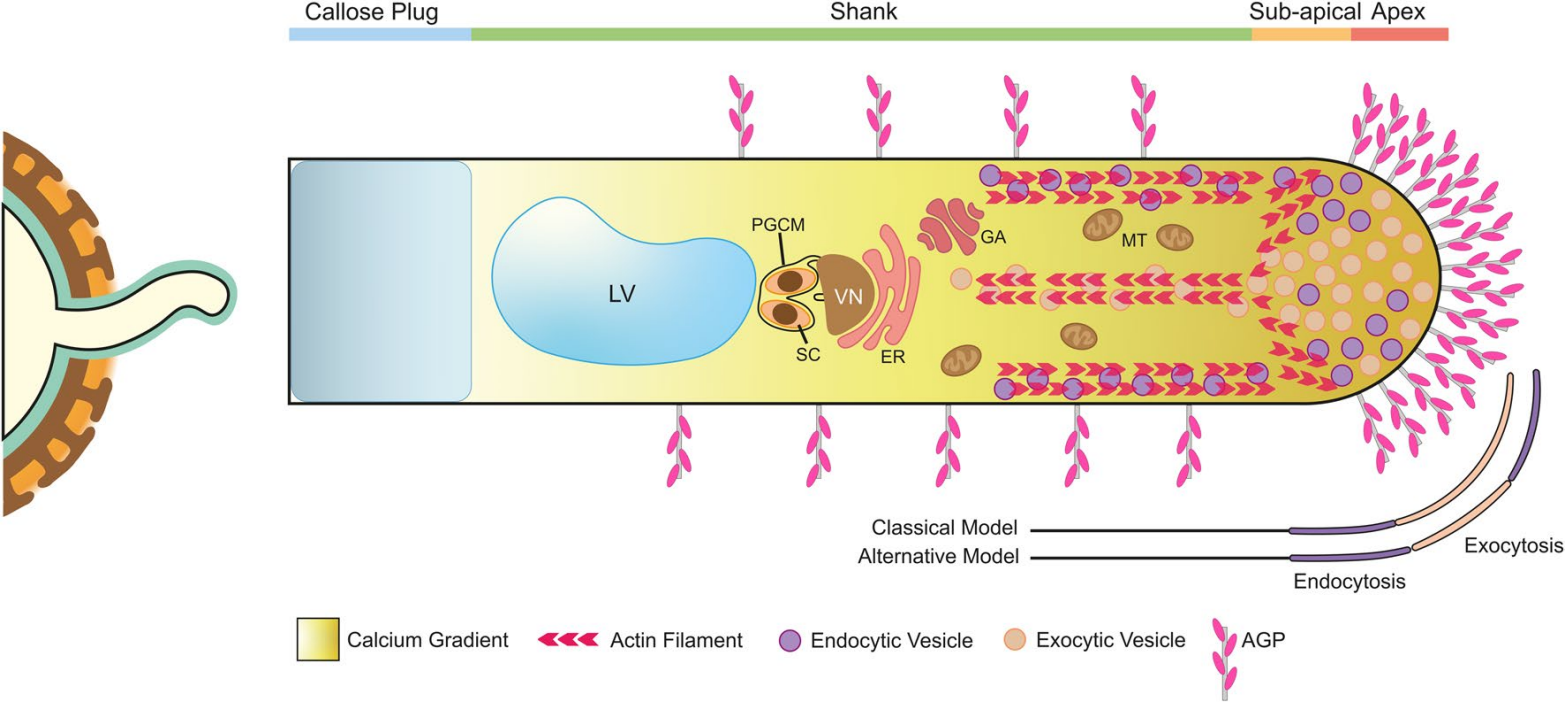
Diagram of the expanding pollen tube tip. The pollen tube (PT) germinates from the aperture site and elongates through tip growth. The PT tip can be divided into the apex, rich in secretory vesicles and AGPs, a sub-apical area where an actin ring resides, and a shank area with lower amount of AGPs compared to the apex and where most organelles dwell, including the endoplasmic reticulum (ER), Golgi apparatus (GA), mitochondria (MT) and a large vacuole (LV) at the rear of the sperm cells (SC), which are enclosed by the peri-germ cell membrane (PGCM) and guided by the vegetative nucleus (VN). Additionally, actin filaments are present in the shank in a parallel array, consisting of a bundle in the centre and one around the edges near the cell wall. The arrangement of actin filaments forms a path for vesicles and possibly organelles, with those at the borders directing them in the direction of the apex, whereas those at the centre form the opposite, creating a reversed fountain flow. Two models of membrane recycling have been proposed: the classical model, in which membrane components are retrieved from the sub-apical region and deposited at the apex, and the alternative model, in which endocytosis occurs at the tip of the apex and sub-apical region and deposition is limited to the flanks of the apex. A callose plug is formed between the tip and the rest of the PT to prevent cellular content retreat, which also plays a fundamental role in relieving excess compression and tensile stress at the tip. Alongside actin filaments, a strong calcium gradient toward the apex aids organelles and secretory vesicles in determining their location. Abbreviations: ER, endoplasmic reticulum; GA, Golgi apparatus; LV, large vacuole; MT, mitochondria; PGCM, peri-germ cell membrane; SC, sperm cells; VN, vegetative nucleus

The number of ovules is small compared to the thousands of PGs released by each anther. Consequently, this process is simultaneously a race between different PTs, which aim to deliver their precious cargo faster. This race depends mostly on how fast a PT can grow, which is dependent not only on the stored nutritional reserves but also on a complex machinery that allows PT tip growth and subsequent cell elongation (Fig. 3) (Adhikari et al. 2020b). The expanding PT tip grows as a result of cytoskeleton organisation, vesicular trafficking, GTPase signalling, and ion gradients. The tip can be divided into three zones: apical, subapical, and shank zones. The apical area, or apex, is filled with secretory vesicles, forming a cone shape, whereas in the subapical area, an actin ring is formed (Guan et al. 2013; Grebnev et al. 2017; Adhikari et al. 2020b; Hafidh and Honys 2021). The shank area is rich in ER, Golgi, and mitochondria, and contains a large vacuole in a basal position (Motomura et al. 2021). Actin filaments may also be found in the shank area but in a parallel array consisting of a bundle in the centre and one around the edges near the cell wall. This arrangement creates an organised pathway for vesicles and maybe also organelles. Whereas those at the edges guide vesicles towards the apical region, those at the centre guide them in the opposite direction, creating a reversed fountain flow. This flow allows the recycling of membrane materials, presumably retrieving them from the subapical area via endocytosis and incorporating them at the apex via exocytosis. This classical model has been challenged and an alternative method was suggested, in which endocytosis, in addition to occurring at the subapical area, would also occur at the apex, while exocytosis becomes restricted to the edges of the apex (Zonia and Munnik 2008). Alongside actin filaments, a strong Ca^2+^ and pH gradient facilitate the positioning of organelles and secretory vesicles within this constantly changing cellular environment, with higher concentrations of Ca^2+^ and H^+^ ions localised at the apex. The cytoplasmic Ca^2+^ gradient may also enhance the secretion of wall materials into the membrane, thereby promoting exocytosis and subsequent vesicle fusion. Consequently, the Ca^2+^ gradient can also control the direction of growth by controlling secretion. However, Ca^2+^ concentration does not appear to influence growth rate (Hepler et al. 2013; Guan et al. 2013; Grebnev et al. 2017; Zheng et al. 2019; Hafidh and Honys 2021).

PT growth is also influenced by the cell wall composition. The PT cell wall has a compositional contrast between the apex and shank areas because of their different functions. In the apex, methylesterified pectins constitute the main component of the cell wall, granting plasticity to this area, which is fundamental to ensure new membrane integration and consequently PT growth. This area also presents AGPs and cellulose. In contrast, the cell wall in older areas, namely the shank, must be more rigid to withstand physical pressure. In this area, the cell wall is bilayered, with an inner layer mostly composed of callose and an outer layer mostly composed of demethylesterified pectin. Additionally, the shank cell wall contains lower amounts of cellulose and AGPs (Dardelle et al. 2010). The shift of methylesterified pectin in the apex to demethylesterified pectin in the shank is due to the activity of pectin methylesterases, which are capable of de-esterification of homogalacturonan residues and modification of methoxyl groups into carboxyl groups. Owing to their negative charge, carboxyl groups cross-link with Ca^2+^, forming a firmer pectin network. This process does not occur at the apex because of the presence of pectin methylesterase inhibitors in this area, which are removed through endocytosis once they reach the subapical area, allowing pectin methylesterases to alter the pectin present. Another key component of the PT wall is the callose plugs deposited at regular distances which relieve the PT tip of excess compression and tensile stress. Callose plugs also act as physical obstructions to the backward flow of organelles (Chebli et al. 2012; Hepler et al. 2013; Adhikari et al. 2020a; Hao et al. 2022).

The β-Yariv reagent binds to and precipitates most AGPs and has thus been widely used to infer AGP function as a whole (Kitazawa et al. 2013). In *N. tabacum*, the use of β-Yariv reagent led to slower growth of PTs (Qin et al. 2007), and in *Fragaria x ananassa*, PT growth was inhibited after one hour in medium containing β-Yariv reagent. When transferred to fresh medium, new PTs formed after two hours but these were stiffer than expected, and the brief “neutralisation” of AGPs led to an absence of cellulose in the apex or an atypical ring-like cellulose formation along the PT. Furthermore, callose deposition was altered since PTs treated with β-Yariv reagent showed callose throughout the PT or in the shank and tip (Leszczuk et al. 2019). In *Asimina triloba,* β-Yariv reagent was added to the medium 3 h after PTs started germinating, and as a consequence their growth rate and metabolic rate decreased (Losada et al. 2017). β-Yariv reagent has been used on PTs of *A. thaliana* and shown to change PT shape, as well as resulting in growth arrest and alteration in F-actin organisation, especially at the apex and sub-apical region. Furthermore, *O*-glycosylation disruption of HRGPs caused PT branching, bulging, and alterations in F-actin positioning (Lara-Mondragón et al. 2022).

## AGPs sugars in PG development and PT growth

Recently, the role of AGPs in plant development has been studied by targeting specific glycosyltransferases that add sugar moieties to AGPs. Several studies have highlighted the importance of Hyp-*O*-galactosyltransferases (GALTs), namely GALT2 (AT4G21060), GALT3 (AT3G06440), GALT4 (AT1G27120), GALT5 (AT1G74800), GALT6 (AT5G62620), GALT7 (AT5G53340), GALT8 (AT4G32120), and GALT9 (AT2G25300), which are enzymes that add the first galactose residue to Hyp residues in the AGP peptide backbone. Several single to octuple mutants of these GALT genes revealed a lower seed set compared to wt (Kaur et al. 2021, 2022, 2023; Moreira et al. 2023, 2024). A quintuple *galt2 galt5 galt7 galt8 galt9* and octuple *galt2 galt3 galt4 galt5 galt6 galt7 galt8 galt9* mutants showed reduced anthers and PG germination rates, as well as reduced PG release. Furthermore, after meiosis, the tapetum cells of the *galt2 galt5 galt7 galt8 galt9* mutant were enlarged and heavily vacuolated. Correspondingly, microspores were also shown to have a large number of lytic vacuoles, which was theorised to be due to an increase in metabolic rates, and mutant PGs did not undergo mitosis and could not form an intine layer. This coupled with an unusual exine structure with small or hampered lacunae and an unconventional reticulated structure, resulted in the collapse of approximately 28% of PGs. Developing PGs had a less developed intine, and the exine detached with ease which, along with the remains of collapsed PGs, made them adhere to each other, causing the release defect. Regarding the PT, the mutations caused a reduction in length, probably because of defective callose distribution, consisting of an excess at the tip (Kaur et al. 2021, 2022). This coincides with the presence of AGPs at the tip of PTs (Pereira et al. 2006). These abnormalities were only amplified in the octuple *galt2 galt3 galt4 galt5 galt6 galt7 galt8 galt9* mutant in which PG collapse reached 66.7%. Moreover, PGs often had no nuclei and anthers had a developmental delay, especially concerning dehiscence. Both microspores and the anther endothecium wall showed an aggravated callose deposition and the formation of large lytic vacuoles started in PMCs. PG germination in vitro decreased 71% compared to wt (Kaur et al. 2023).

Similarly, a null mutant of *KNS4*/*UPEX1* (AT1G33430), a galactosyltransferase associated with type II arabinogalactan biosynthesis, exhibited aberrations in PGs. The mutant PGs displayed a collapsed morphology with reduced nuclei, along with instances of normal PGs adhering to each other and to anther tissues, which was attributed in part to debris. The PMC stage marked the onset of the observed defects. Mutant PMCs remained adhered to each other and to tapetal cells, which were larger and more vacuolated than expected, thereby exerting pressure on the developing microspores (Suzuki et al. 2017). Likewise, tetrads exhibited adhesion abnormalities due to remnant callose, affecting proper exine development. This manifested as a thinner primexine, resulting in shorter probaculae/baculae, undefined tectum, and a lesser amount of pollen coat, due to challenges in anchoring primexine to the microspore (Li et al. 2017; Suzuki et al. 2017). In addition to abnormal AGP distribution in later stages (above the tectum instead of between baculae), the authors also observed a difference in low- and unesterified pectin, which formed a layer on the nexine layer. Additionally, PT elongation was deficient compared to that of wt, which was associated with fewer seeds and shorter siliques (Suzuki et al. 2017).

Likewise, glucuronosyltransferases (GLCATs), responsible for adding glucuronic acid to β-1,6- and β-1,3-galactose chains of AGPs, have recently become the focus of genetic manipulation aimed at unravelling AGP function. Several mutants targeting GLCAT14A (AT5G39990), GLCAT14B (AT5G15050), GLCAT14C (AT2G37585), GLCAT14D (AT3G24040), and GLCAT14E (AT3G15350) have been successfully characterised (Zhang et al. 2020; Ajayi et al. 2021, 2022). Mutants such as *glcat14a glcat14b* and *glcat14a glcat14b glcat14c* exhibited defective PGs, with the latter displaying a lower PG germination percentage but a normal PT length. The defective PGs were attributed to an abnormal intine and exine development with fewer bacula. Additionally, these mutants had reduced Ca^2+^ binding capacity compared to wt and showed defects in polytube blocking (Zhang et al. 2020; Ajayi et al. 2022). Furthermore, *glcat14b glacat14c* and *glcat14a glcat14b glcat14c* mutants had shorter siliques due to a decrease in seed set (Zhang et al. 2020).

## Mode of action of AGPs

AGPs have been found throughout the plant kingdom, from bryophytes to angiosperms, and even in algae, indicating a strong evolutionary necessity for their presence and appear to be almost ubiquitous in plant developmental processes (Ma et al. 2018). However, the precise mechanism underlying their mode of action remains unclear. Several theories have been hypothesised to explain how AGPs operate at the molecular level; however, since AGPs are a large group of proteins, several functional mechanisms might be involved. For instance, Lamport and Várnai (2013) suggested that AGPs function as Ca^2+^ capacitors, implying that AGPs are involved in Ca^2+^-dependent signalling pathways at the plasma membrane level. The capacity of AGPs to bind to Ca^2+^ was proposed to be due to the presence of 4-*O*-methyl glucuronic acid residues in their sugar chains. This theory was further supported by a recent study by Lopez-Hernandez et al. (2020), who determined that AGPs were important in signalling networks by providing a source of apoplastic Ca^2+^ in a pH-dependent manner. PT growth has been extensively used by Lamport and collaborators (Lamport and Várnai 2013; Lamport et al. 2018) to explain how AGPs may influence Ca^2+^ signalling pathways, leading to cell expansion. This phenomenon was postulated to exhibit oscillatory behaviour in which the elongating PT initiates the opening of stretch-activated H^+^-ATPases, leading to a pH decrease in the apoplast. Acidification of the apoplast causes the dissociation of Ca^2+^ ions from AGPs. Subsequently, through the activation of stretch-activated Ca^2+^ channels, Ca^2+^ enters into the cytosol at the apex of the growing PT. The Ca^2+^ ions released into the cytosol are brought back in vesicles that fuse with the plasma membrane, facilitating PT elongation. As the pH in the apoplast increases, AGPs bind to the returning Ca^2+^, completing the cycle.

Another signalling pathway in which AGPs might be important is related to the cleavage of their GPI-anchor by phospholipase C (Olmos et al. 2017; Hromadová et al. 2021). This possible signalling pathway was observed in in vitro salt-adapted tobacco cells, where AGPs accumulated extracellularly. It was theorised that AGPs bound to the plasma membrane had their GPI-anchor cleaved and freed to the extracellular space to regulate cell wall extensibility by acting as pectin plasticisers, thus assisting cell growth under osmotic stress. Furthermore, GPI-anchored AGPs may be able to transport sodium (Na^+^) ions to vacuoles by tendering them to endocytic vesicles that later fuse with the tonoplast. Na^+^ binding to AGPs would occur through a mechanism similar to that of Ca^2+^, involving glucuronidation of AG residues and enabling them to sequester superfluous cytosolic Na^+^ in saline environments (Olmos et al. 2017). This means that AGPs may function as membrane receptors, as well as ion capacitors.

A comprehensive review of the implication of AGPs in cellulose synthesis and deposition pattern was recently published by Lin et al. (2022), where several mechanisms for AGP action were proposed: (A) similar to Ca^2+^ binding, AG residues of GPI-anchored AGPs could sense external signals and transmit them to the plasma membrane-bound receptor kinases and, through an ethylene-independent 1-aminocyclopropane-1-carboxylic acid pathway, promote cellulose synthesis. However, the authors pointed out that other pathways are able to promote cellulose synthesis in which AG residues still bind to molecular signals; (B) similarly, cleaved AG residues could act as signal molecules to transmembrane proteins, thus participating in the cellulose synthesis pathway; (C) by associating with other cell wall components such as pectin and hemicellulose, as well as eventually structural proteins or even cellulose itself, AGPs could affect cellulose deposition; (D) AGPs may be able to regulate the deposition pattern of cellulose in conjunction with transmembrane proteins affecting the arrangement or connection of cortical microtubules necessary for cellulose synthesis; (E) in conjunction with the hypothesis provided by Lamport et al. (2018), Lin et al. (2022) suggested that AGPs, serving as Ca^2+^ binding proteins, could create cross-links with pectin-Ca^2+^, which, in turn, could regulate cellulose deposition in the cell wall. Likewise, Mi et al. (2022) speculated that intine located AGPs bound to pectin residues enforce correct cellulose positioning, and that if a defect occurs in AGP deposition, this will cause a chain reaction leading to poor cellulose deposition that causes intine abnormalities, eventually leading to programmed cell death and PG abortion due to reactive oxygen species (ROS) signalling. In support of theories (C) and (D) provided by Lin et al. (2022), Tan et al. (2013, 2023) demonstrated the existence of an AGP covalently bonded to pectin through rhamnose residues present in their AG chains, whereas their rhamnose or arabinose residues can link to xylan.

## Conclusion and future perspectives

In summary, AGPs are involved in pollen development from the PMC stage to the growth and guidance of PTs. AGPs appear to be mostly involved in nexine and intine formation, being key elements in primexine anchoring as well as cellulose deposition. Moreover, AGPs were also found in exine lacunae, around aperture sites, and in the subapical and apical areas of the PT. A lack of AGPs usually leads to the collapse of PGs at the bicellular microspore stage and pollen mitosis arrest. In PTs, AGPs appear to be essential for proper growth, cellulose and callose deposition, as well as F-actin positioning. Therefore, AGPs play key roles in plant sexual reproduction, including pollen development, germination, PT growth, and interaction with female reproductive tissues. Understanding the functions of AGPs in pollen can provide insights into plant reproductive biology and may have implications for crop improvement and plant breeding strategies.

We propose that not only are AGPs a major component of the nexine layer, as well as essential in primexine anchoring, and thus correct exine formation, but also a main component of either exintine or the space between the intine and the plasma membrane. AGPs, especially FLAs, may cross-link with pectin, influencing the cellulose deposition process in the endintine. The lack of structural support in the microspore, caused by a lack of AGPs, creates an unstable internal environment, which leads to the collapse of the cytoplasm before the second pollen mitosis is complete. However, it is worth mentioning that the lack of AGPs may also disturb the vacuolation process, which is fundamental for pollen mitosis.

Although AGPs in pollen grain development and pollen tube growth have been extensively studied, their precise mode of action remains largely theoretical, despite recent advances in the field. Current theories predominantly concentrate on the role of AGPs in the elongation of the PT, yet the broader role of AGPs during the early stages of pollen development remains elusive. Several key questions warrant further investigation to provide a comprehensive understanding of AGPs’ functions. Are AGPs integral components of the primexine layer, or are they more closely associated with the plasma membrane, thus anchoring primexine components through their interactions? Understanding their precise localisation could reveal how AGPs contribute to the structural integrity and developmental processes of the pollen wall. What role do AGPs play in cellulose deposition during PG development? Investigating how AGPs interact with cellulose synthase complexes or other components involved in cellulose biosynthesis could elucidate their role in cell wall formation and stabilisation. Are AGPs incorporated into the intine layer of the pollen wall, or do they play a role in linking this layer to the plasma membrane? Clarifying this could enhance our understanding of how the intine layer is organised and maintained. If AGPs are indeed present in significant quantities in the nexine layer, as previously suggested, what specific functions do they perform there? Delving into their role could provide insights into how AGPs contribute to the structural and functional properties of this crucial pollen wall layer. How does the dissociation of AGPs from Ca^2+^ influence PG development? Given the critical role of Ca^2+^ in cell signalling and developmental processes, understanding the relationship between AGPs and Ca^2+^ dynamics could shed light on how AGPs regulate pollen development through ionic interactions. To what extent are AGPs involved in the organisation of F-actin during PT growth? Since F-actin plays a pivotal role in PT elongation and morphogenesis, exploring how AGPs influence F-actin dynamics could reveal their contributions to the growth and guidance of the pollen tube. Addressing these questions will not only advance our knowledge of AGPs in pollen biology but also potentially uncover new mechanisms underlying pollen development and fertilisation processes. Continued research in these areas is crucial for a deeper understanding of AGPs' roles and could have broader implications for plant reproductive biology and crop improvement strategies.

## References

[CR1] Adhikari PB, Liu X, Kasahara RD (2020a) Mechanics of pollen tube elongation: a perspective. Front Plant Sci 11:589712. 10.3389/fpls.2020.589712 33193543 10.3389/fpls.2020.589712 PMC7606272

[CR2] Adhikari PB, Liu X, Wu X et al (2020b) Fertilization in flowering plants: an odyssey of sperm cell delivery. Plant Mol Biol 103:9–32. 10.1007/s11103-020-00987-z 32124177 10.1007/s11103-020-00987-z

[CR3] Ajayi OO, Held MA, Showalter AM (2021) Three β-glucuronosyltransferase genes involved in arabinogalactan biosynthesis function in Arabidopsis growth and development. Plants 10:1172. 10.3390/plants10061172 34207602 10.3390/plants10061172 PMC8227792

[CR4] Ajayi OO, Held MA, Showalter AM (2022) Glucuronidation of type II arabinogalactan polysaccharides function in sexual reproduction of Arabidopsis. Plant J 109:164–181. 10.1111/tpj.15562 34726315 10.1111/tpj.15562

[CR5] Anand S, Tyagi AK (2010) Characterization of a pollen-preferential gene OSIAGP from rice (*Oryza sativa* L. subspecies *indica*) coding for an arabinogalactan protein homologue, and analysis of its promoter activity during pollen development and pollen tube growth. Transgenic Res 19:385–397. 10.1007/s11248-009-9319-3 19771527 10.1007/s11248-009-9319-3

[CR6] Borg M, Brownfield L, Twell D (2009) Male gametophyte development: a molecular perspective. J Exp Bot 60:1465–1478. 10.1093/jxb/ern355 19213812 10.1093/jxb/ern355

[CR7] Chebli Y, Kaneda M, Zerzour R, Geitmann A (2012) The cell wall of the Arabidopsis pollen tube—spatial distribution, recycling, and network formation of polysaccharides. Plant Physiol 160:1940–1955. 10.1104/pp.112.199729 23037507 10.1104/pp.112.199729 PMC3510122

[CR8] Coimbra S, Almeida J, Junqueira V et al (2007) Arabinogalactan proteins as molecular markers in *Arabidopsis thaliana* sexual reproduction. J Exp Bot. 10.1093/jxb/erm259 18039740 10.1093/jxb/erm259

[CR9] Coimbra S, Jones B, Pereira LG (2008) Arabinogalactan proteins (AGPs) related to pollen tube guidance into the embryo sac in Arabidopsis. Plant Signal Behav 3:455–456. 10.4161/psb.3.7.5601 19704483 10.4161/psb.3.7.5601 PMC2634427

[CR10] Coimbra S, Costa M, Jones B et al (2009) Pollen grain development is compromised in *Arabidopsis agp6 agp11* null mutants. J Exp Bot 60:3133–3142. 10.1093/jxb/erp148 19433479 10.1093/jxb/erp148 PMC2718217

[CR11] Coimbra S, Costa M, Mendes MA et al (2010) Early germination of *Arabidopsis* pollen in a double null mutant for the arabinogalactan protein genes *AGP6* and *AGP11*. Sex Plant Reprod 23:199–205. 10.1007/s00497-010-0136-x 20162305 10.1007/s00497-010-0136-x

[CR12] Corral-Martínez P, Driouich A, Seguí-Simarro JM (2019) Dynamic changes in arabinogalactan-protein, pectin, xyloglucan and xylan composition of the cell wall during microspore embryogenesis in *Brassica napus*. Front Plant Sci 10:332. 10.3389/fpls.2019.00332 30984213 10.3389/fpls.2019.00332 PMC6447685

[CR13] Costa M, Pereira AM, Rudall PJ, Coimbra S (2013) Immunolocalization of arabinogalactan proteins (AGPs) in reproductive structures of an early-divergent angiosperm, *Trithuria* (Hydatellaceae). Ann Bot 111:183–190. 10.1093/aob/mcs256 23186834 10.1093/aob/mcs256 PMC3555524

[CR14] Costa ML, Sobral R, Costa MMR et al (2015) Evaluation of the presence of arabinogalactan proteins and pectins during *Quercus suber* male gametogenesis. Ann Bot 115:81–92. 10.1093/aob/mcu223 25452249 10.1093/aob/mcu223 PMC4284116

[CR15] Dardelle F, Lehner A, Ramdani Y et al (2010) Biochemical and immunocytological characterizations of Arabidopsis pollen tube cell wall. Plant Physiol 153:1563–1576. 10.1104/pp.110.158881 20547702 10.1104/pp.110.158881 PMC2923879

[CR16] Dumas C, Knox RB, Gaude T (1985) The spatial association of the sperm cells and vegetative nucleus in the pollen grain of *Brassica*. Protoplasma 124:168–174. 10.1007/BF01290767

[CR17] El-Tantawy A-A, Solís M-T, Da Costa ML et al (2013) Arabinogalactan protein profiles and distribution patterns during microspore embryogenesis and pollen development in *Brassica napus*. Plant Reprod 26:231–243. 10.1007/s00497-013-0217-8 23729197 10.1007/s00497-013-0217-8

[CR18] Gómez JF, Talle B, Wilson ZA (2015) Anther and pollen development: a conserved developmental pathway. J Integr Plant Biol 57:876–891. 10.1111/jipb.12425 26310290 10.1111/jipb.12425 PMC4794635

[CR19] Grebnev G, Ntefidou M, Kost B (2017) Secretion and endocytosis in pollen tubes: models of tip growth in the spot light. Front Plant Sci 8:154. 10.3389/fpls.2017.00154 28224002 10.3389/fpls.2017.00154 PMC5293803

[CR20] Guan Y, Guo J, Li H, Yang Z (2013) Signaling in pollen tube growth: crosstalk, feedback, and missing links. Mol Plant 6:1053–1064. 10.1093/mp/sst070 23873928 10.1093/mp/sst070 PMC3842152

[CR21] Hafidh S, Honys D (2021) Reproduction multitasking: the male gametophyte. Annu Rev Plant Biol 72:581–614. 10.1146/annurev-arplant-080620-021907 33900787 10.1146/annurev-arplant-080620-021907

[CR22] Halbritter H, Ulrich S, Grímsson F et al (2018) Illustrated pollen terminology. Springer, Cham

[CR23] Hao G-J, Zhao X-Y, Zhang M-M et al (2022) Vesicle trafficking in *Arabidopsis* pollen tubes. FEBS Lett 596:2231–2242. 10.1002/1873-3468.14343 35348201 10.1002/1873-3468.14343

[CR24] Hater F, Nakel T, Groß-Hardt R (2020) Reproductive multitasking: the female gametophyte. Annu Rev Plant Biol 71:517–546. 10.1146/annurev-arplant-081519-035943 32442389 10.1146/annurev-arplant-081519-035943

[CR25] Hepler PK, Rounds CM, Winship LJ (2013) Control of cell wall extensibility during pollen tube growth. Mol Plant 6:998–1017. 10.1093/mp/sst103 23770837 10.1093/mp/sst103 PMC4043104

[CR26] Hromadová D, Soukup A, Tylová E (2021) Arabinogalactan proteins in plant roots – an update on possible functions. Front Plant Sci 12:674010. 10.3389/fpls.2021.674010 34079573 10.3389/fpls.2021.674010 PMC8165308

[CR27] Huang H, Miao Y, Zhang Y et al (2021) Comprehensive analysis of arabinogalactan protein-encoding genes reveals the involvement of three *BrFLA* genes in pollen germination in *Brassica rapa*. Int J Mol Sci 22:13142. 10.3390/ijms222313142 34884948 10.3390/ijms222313142 PMC8658186

[CR28] Jia QS, Zhu J, Xu XF et al (2015) *Arabidopsis* AT-hook protein TEK positively regulates the expression of arabinogalactan proteins for nexine formation. Mol Plant 8:251–260. 10.1016/j.molp.2014.10.001 25616387 10.1016/j.molp.2014.10.001

[CR29] Jiang J, Zhang Z, Cao J (2013) Pollen wall development: the associated enzymes and metabolic pathways. Plant Biol 15:249–263. 10.1111/j.1438-8677.2012.00706.x 23252839 10.1111/j.1438-8677.2012.00706.x

[CR30] Johnson KL, Cassin AM, Lonsdale A et al (2017) A motif and amino acid bias bioinformatics pipeline to identify hydroxyproline-rich glycoproteins. Plant Physiol 174:886–903. 10.1104/pp.17.00294 28446635 10.1104/pp.17.00294 PMC5462032

[CR31] Kaur D, Held MA, Smith MR, Showalter AM (2021) Functional characterization of hydroxyproline-*O*-galactosyltransferases for Arabidopsis arabinogalactan-protein synthesis. BMC Plant Biol 21:1–24. 10.1186/S12870-021-03362-2 34903166 10.1186/s12870-021-03362-2 PMC8667403

[CR32] Kaur D, Moreira D, Coimbra S, Showalter AM (2022) Hydroxyproline-*O*-galactosyltransferases synthesizing type II arabinogalactans are essential for male gametophytic development in Arabidopsis. Front Plant Sci 13:935413. 10.3389/fpls.2022.935413 35774810 10.3389/fpls.2022.935413 PMC9237623

[CR33] Kaur D, Held MA, Zhang Y et al (2023) Knockout of eight *hydroxyproline-O-galactosyltransferases* cause multiple vegetative and reproductive growth defects. Cell Surf 10:100117. 10.1016/j.tcsw.2023.100117 38076635 10.1016/j.tcsw.2023.100117 PMC10698532

[CR34] Kitazawa K, Tryfona T, Yoshimi Y et al (2013) β-galactosyl Yariv reagent binds to the β-1,3-galactan of arabinogalactan proteins. Plant Physiol 161:1117–1126. 10.1104/pp.112.211722 23296690 10.1104/pp.112.211722 PMC3585584

[CR35] Lamport DTA, Várnai P (2013) Periplasmic arabinogalactan glycoproteins act as a calcium capacitor that regulates plant growth and development. New Phytol 197:58–64. 10.1111/nph.12005 23106282 10.1111/nph.12005

[CR36] Lamport DTA, Tan L, Held MA, Kieliszewski MJ (2018) Pollen tube growth and guidance: Occam’s razor sharpened on a molecular arabinogalactan glycoprotein Rosetta Stone. New Phytol 217:491–500. 10.1111/nph.14845 28990197 10.1111/nph.14845

[CR37] Lara-Mondragón CM, Dorchak A, MacAlister CA (2022) *O*-glycosylation of the extracellular domain of pollen class I formins modulates their plasma membrane mobility. J Exp Bot 73:3929–3945. 10.1093/jxb/erac131 35383367 10.1093/jxb/erac131 PMC9232206

[CR38] Leszczuk A, Kozioł A, Szczuka E, Zdunek A (2019) Analysis of AGP contribution to the dynamic assembly and mechanical properties of cell wall during pollen tube growth. Plant Sci 281:9–18. 10.1016/j.plantsci.2019.01.005 30824065 10.1016/j.plantsci.2019.01.005

[CR39] Levitin B, Richter D, Markovich I, Zik M (2008) Arabinogalactan proteins 6 and 11 are required for stamen and pollen function in Arabidopsis. Plant J 56:351–363. 10.1111/j.1365-313X.2008.03607.x 18644001 10.1111/j.1365-313X.2008.03607.x

[CR40] Li J, Yu M, Geng LL, Zhao J (2010) The fasciclin-like arabinogalactan protein gene, *FLA3*, is involved in microspore development of Arabidopsis. Plant J 64:482–497. 10.1111/j.1365-313X.2010.04344.x 20807209 10.1111/j.1365-313X.2010.04344.x

[CR41] Li WL, Liu Y, Douglas CJ (2017) Role of glycosyltransferases in pollen wall primexine formation and exine patterning. Plant Physiol 173:167–182. 10.1104/pp.16.00471 27495941 10.1104/pp.16.00471 PMC5210704

[CR42] Lin S, Dong H, Zhang F et al (2014) *BcMF8*, a putative arabinogalactan protein-encoding gene, contributes to pollen wall development, aperture formation and pollen tube growth in *Brassica campestris*. Ann Bot 113:777–788. 10.1093/aob/mct315 24489019 10.1093/aob/mct315 PMC3962243

[CR43] Lin S, Yue X, Miao Y et al (2018) The distinct functions of two classical arabinogalactan proteins BcMF8 and BcMF18 during pollen wall development in *Brassica campestris*. Plant J 94:60–76. 10.1111/tpj.13842 29385650 10.1111/tpj.13842

[CR44] Lin S, Huang L, Miao Y et al (2019) Constitutive overexpression of the classical arabinogalactan protein gene *BcMF18* in *Arabidopsis* causes defects in pollen intine morphogenesis. Plant Growth Regul 88:159–171. 10.1007/s10725-019-00496-0

[CR45] Lin S, Miao Y, Huang H et al (2022) Arabinogalactan proteins: focus on the role in cellulose synthesis and deposition during plant cell wall biogenesis. Int J Mol Sci 23:6578. 10.3390/ijms23126578 35743022 10.3390/ijms23126578 PMC9223364

[CR46] Lopes AL, Moreira D, Ferreira MJ et al (2019) Insights into secrets along the pollen tube pathway in need to be discovered. J Exp Bot 70:2979–2992. 10.1093/jxb/erz087 30820535 10.1093/jxb/erz087

[CR47] Lopez-Hernandez F, Tryfona T, Rizza A et al (2020) Calcium binding by arabinogalactan polysaccharides is important for normal plant development. Plant Cell 32:3346–3369. 10.1105/tpc.20.00027 32769130 10.1105/tpc.20.00027 PMC7534474

[CR48] Losada JM, Hormaza JI, Lora J (2017) Pollen–pistil interaction in pawpaw (*Asimina triloba*), the northernmost species of the mainly tropical family Annonaceae. Am J Bot 104:1891–1903. 10.3732/ajb.1700319 29217674 10.3732/ajb.1700319

[CR49] Lou Y, Xu X-F, Zhu J et al (2014a) The tapetal AHL family protein TEK determines nexine formation in the pollen wall. Nat Commun 5:3855. 10.1038/ncomms4855 24804694 10.1038/ncomms4855 PMC4024750

[CR50] Lou Y, Zhu J, Yang Z (2014b) Molecular cell biology of pollen walls. In: Nick P, Opatrny Z (eds) Applied plant cell biology: cellular tools and approaches for plant biotechnology. Springer, Berlin, pp 179–205

[CR51] Ma Y, Yan C, Li H et al (2017) Bioinformatics prediction and evolution analysis of arabinogalactan proteins in the plant kingdom. Front Plant Sci. 10.3389/fpls.2017.00066 28184232 10.3389/fpls.2017.00066 PMC5266747

[CR52] Ma Y, Zeng W, Bacic A, Johnson K (2018) AGPs through time and space. Annu Plant Rev Online 1:1–38. 10.1002/9781119312994.apr0608

[CR53] Ma T, Dong F, Luan D et al (2019) Gene expression and localization of arabinogalactan proteins during the development of anther, ovule, and embryo in rice. Protoplasma 256:909–922. 10.1007/s00709-019-01349-3 30675653 10.1007/s00709-019-01349-3

[CR54] McCue AD, Cresti M, Feijó JA, Slotkin RK (2011) Cytoplasmic connection of sperm cells to the pollen vegetative cell nucleus: potential roles of the male germ unit revisited. J Exp Bot 62:1621–1631. 10.1093/jxb/err032 21357775 10.1093/jxb/err032

[CR55] Mi L, Mo A, Yang J et al (2022) Arabidopsis Novel Microgametophyte Defective Mutant 1 is required for pollen viability *via* influencing intine development in Arabidopsis. Front Plant Sci 13:814870. 10.3389/fpls.2022.814870 35498668 10.3389/fpls.2022.814870 PMC9039731

[CR56] Miao Y, Cao J, Huang L et al (2021) *FLA14* is required for pollen development and preventing premature pollen germination under high humidity in Arabidopsis. BMC Plant Biol 21:254. 10.1186/s12870-021-03038-x 34082704 10.1186/s12870-021-03038-x PMC8173729

[CR57] Moreira D, Lopes AL, Silva J et al (2022) New insights on the expression patterns of specific Arabinogalactan proteins in reproductive tissues of *Arabidopsis thaliana*. Front Plant Sci 13:1083098. 10.3389/fpls.2022.1083098 36531351 10.3389/fpls.2022.1083098 PMC9755587

[CR58] Moreira D, Kaur D, Pereira AM et al (2023) Type II arabinogalactans initiated by hydroxyproline-O-galactosyltransferases play important roles in pollen–pistil interactions. Plant J 114:371–389. 10.1111/tpj.16141 36775989 10.1111/tpj.16141

[CR59] Moreira D, Kaur D, Fourbert-Mendes S et al (2024) Eight hydroxyproline-*O*-galactosyltransferases play essential roles in female reproductive development. Plant Sci 348:112231. 10.1016/j.plantsci.2024.112231 39154893 10.1016/j.plantsci.2024.112231

[CR60] Motomura K, Takeuchi H, Notaguchi M et al (2021) Persistent directional growth capability in *Arabidopsis thaliana* pollen tubes after nuclear elimination from the apex. Nat Commun 12:2331. 10.1038/s41467-021-22661-8 33888710 10.1038/s41467-021-22661-8 PMC8062503

[CR61] Nguema-Ona E, Coimbra S, Vicré-Gibouin M et al (2012) Arabinogalactan proteins in root and pollen-tube cells: distribution and functional aspects. Ann Bot 110:383–404. 10.1093/aob/mcs143 22786747 10.1093/aob/mcs143 PMC3394660

[CR62] Olmos E, García De La Garma J, Gomez-Jimenez MC, Fernandez-Garcia N (2017) Arabinogalactan proteins are involved in salt-adaptation and vesicle trafficking in tobacco BY-2 cell cultures. Front Plant Sci 8:1092. 10.3389/fpls.2017.01092 28676820 10.3389/fpls.2017.01092 PMC5476920

[CR63] Pereira LG, Coimbra S, Oliveira H et al (2006) Expression of arabinogalactan protein genes in pollen tubes of *Arabidopsis thaliana*. Planta 223:374–380. 10.1007/s00425-005-0137-4 16228244 10.1007/s00425-005-0137-4

[CR64] Pereira AM, Masiero S, Nobre MS et al (2014) Differential expression patterns of arabinogalactan proteins in *Arabidopsis thaliana* reproductive tissues. J Exp Bot 65:5459–5471. 10.1093/jxb/eru300 25053647 10.1093/jxb/eru300 PMC4400541

[CR65] Qin Y, Chen D, Zhao J (2007) Localization of arabinogalactan proteins in anther, pollen, and pollen tube of *Nicotiana tabacum* L. Protoplasma 231:43–53. 10.1007/s00709-007-0245-z 17602278 10.1007/s00709-007-0245-z

[CR66] Ruprecht C, Bartetzko MP, Senf D et al (2017) A synthetic glycan microarray enables epitope mapping of plant cell wall glycan-directed antibodies. Plant Physiol 175:1094–1104. 10.1104/pp.17.00737 28924016 10.1104/pp.17.00737 PMC5664464

[CR67] Seifert GJ, Roberts K (2007) The biology of arabinogalactan proteins. Annu Rev Plant Biol 58:137–161. 10.1146/annurev.arplant.58.032806.103801 17201686 10.1146/annurev.arplant.58.032806.103801

[CR68] Shi J, Cui M, Yang L et al (2015) Genetic and biochemical mechanisms of pollen wall development. Trends Plant Sci 20:741–753. 10.1016/j.tplants.2015.07.010 26442683 10.1016/j.tplants.2015.07.010

[CR69] Showalter AM, Keppler B, Lichtenberg J et al (2010) A bioinformatics approach to the identification, classification, and analysis of hydroxyproline-rich glycoproteins. Plant Physiol 153:485–513. 10.1104/pp.110.156554 20395450 10.1104/pp.110.156554 PMC2879790

[CR70] Silva J, Ferraz R, Dupree P et al (2020) Three decades of advances in arabinogalactan-protein biosynthesis. Front Plant Sci 11:2014. 10.3389/fpls.2020.610377 10.3389/fpls.2020.610377 PMC7769824 33384708

[CR71] Sprunck S (2020) Twice the fun, double the trouble: gamete interactions in flowering plants. Curr Opin Plant Biol 53:106–116. 10.1016/j.pbi.2019.11.003 31841779 10.1016/j.pbi.2019.11.003

[CR72] Sugi N, Izumi R, Tomomi S et al (2023) Removal of the endoplasma membrane upon sperm cell activation after pollen tube discharge. Front Plant Sci 14:1116289. 10.3389/fpls.2023.1116289 36778680 10.3389/fpls.2023.1116289 PMC9909283

[CR73] Sugi N, Calhau ARM, Jacquier NMA et al (2024) The peri-germ cell membrane: poorly characterized but key interface for plant reproduction. Nat Plants. 10.1038/s41477-024-01818-5 39406861 10.1038/s41477-024-01818-5 PMC12582372

[CR74] Suzuki T, Narciso JO, Zeng W et al (2017) KNS4/UPEX1: a type II arabinogalactan β-(1,3)-galactosyltransferase required for pollen exine development. Plant Physiol 173:183–205. 10.1104/pp.16.01385 27837085 10.1104/pp.16.01385 PMC5210738

[CR75] Tan H, Liang W, Hu J, Zhang D (2012) *MTR1* encodes a secretory fasciclin glycoprotein required for male reproductive development in rice. Dev Cell 22:1127–1137. 10.1016/j.devcel.2012.04.011 22698279 10.1016/j.devcel.2012.04.011

[CR76] Tan L, Eberhard S, Pattathil S et al (2013) An *Arabidopsis* cell wall proteoglycan consists of pectin and arabinoxylan covalently linked to an arabinogalactan protein. Plant Cell 25:270–287. 10.1105/tpc.112.107334 23371948 10.1105/tpc.112.107334 PMC3584541

[CR77] Tan L, Zhang L, Black I et al (2023) Most of the rhamnogalacturonan-I from cultured Arabidopsis cell walls is covalently linked to arabinogalactan-protein. Carbohydr Polym 301:120340. 10.1016/j.carbpol.2022.120340 36446508 10.1016/j.carbpol.2022.120340

[CR78] Xu H, Knox RB, Taylor PE, Singh MB (1995) *Bcp1*, a gene required for male fertility in *Arabidopsis*. Proc Natl Acad Sci USA 92:2106–2110. 10.1073/pnas.92.6.2106 7892232 10.1073/pnas.92.6.2106 PMC42432

[CR79] Yadegari R, Drews GN (2004) Female gametophyte development. Plant Cell 16:S133–S141. 10.1105/tpc.018192 15075395 10.1105/tpc.018192 PMC2643389

[CR80] Yariv J, Lis H, Katchalski E (1967) Precipitation of arabic acid and some seed polysaccharides by glycosylphenylazo dyes. Biochem J. 10.1042/bj1050001C 6069833 10.1042/bj1050001c PMC1198314

[CR81] Yates EA, Valdor J-F, Haslam SM et al (1996) Characterization of carbohydrate structural features recognized by anti-arabinogalactan-protein monoclonal antibodies. Glycobiology 6:131–139. 10.1093/glycob/6.2.131 8727785 10.1093/glycob/6.2.131

[CR82] Zhang Y, Held MA, Showalter AM (2020) Elucidating the roles of three β-glucuronosyltransferases (GLCATs) acting on arabinogalactan-proteins using a CRISPR-Cas9 multiplexing approach in Arabidopsis. BMC Plant Biol 20:221. 10.1186/s12870-020-02420-5 32423474 10.1186/s12870-020-02420-5 PMC7236193

[CR83] Zhang M, Wei H, Liu J et al (2021) Non-functional *GoFLA19s* are responsible for the male sterility caused by hybrid breakdown in cotton (*Gossypium* spp.). Plant J 107:1198–1212. 10.1111/tpj.15378 34160096 10.1111/tpj.15378

[CR84] Zheng RH, Su SD, Xiao H, Tian HQ (2019) Calcium: a critical factor in pollen germination and tube elongation. Int J Mol Sci 20:420. 10.3390/ijms20020420 30669423 10.3390/ijms20020420 PMC6358865

[CR85] Zieliński K, Dubas E, Gerši Z et al (2021) β-1,3-Glucanases and chitinases participate in the stress-related defence mechanisms that are possibly connected with modulation of arabinogalactan proteins (AGP) required for the androgenesis initiation in rye (Secale cereale L). Plant Science 302:110700. 10.1016/j.plantsci.2020.110700 33288013 10.1016/j.plantsci.2020.110700

[CR86] Zonia L, Munnik T (2008) Vesicle trafficking dynamics and visualization of zones of exocytosis and endocytosis in tobacco pollen tubes. J Exp Bot 59:861–873. 10.1093/jxb/ern007 18304978 10.1093/jxb/ern007

